# A Null Space-Based Blind Source Separation for Fetal Electrocardiogram Signals

**DOI:** 10.3390/s20123536

**Published:** 2020-06-22

**Authors:** Luay Taha, Esam Abdel-Raheem

**Affiliations:** Department of Electrical and Computer Engineering, University of Windsor, 401 Sunset Ave, Windsor, ON N9B 3P4, Canada; eraheem@uwindsor.ca

**Keywords:** fetal electrocardiogram extraction, electrocardiogram peak detection, null space transformation matrix, blind source separation

## Abstract

This paper presents a new non-invasive deterministic algorithm of extracting the fetal Electrocardiogram (FECG) signal based on a new null space idempotent transformation matrix (NSITM). The mixture matrix is used to compute the ITM. Then, the fetal ECG (FECG) and maternal ECG (MECG) signals are extracted from the null space of the ITM. Next, MECG and FECG peaks detection, control logic, and adaptive comb filter are used to remove the unwanted MECG component from the raw FECG signal, thus extracting a clean FECG signal. The visual results from Daisy and Physionet real databases indicate that the proposed algorithm is effective in extracting the FECG signal, which can be compared with principal component analysis (PCA), fast independent component analysis (FastICA), and parallel linear predictor (PLP) filter algorithms. Results from Physionet synthesized ECG data show considerable improvement in extraction performances over other algorithms used in this work, considering different additive signal-to-noise ratio (SNR) increasing from 0 dB to 12 dB, and considering different fetal-to-maternal SNR increasing from −30 dB to 0 dB. The FECG detection of the NSITM is evaluated using statistical measures and results show considerable improvement in the sensitivity (SE), the accuracy (ACC), and the positive predictive value (PPV), as compared with other algorithms. The study demonstrated that the NSITM is a feasible algorithm for FECG extraction.

## 1. Introduction

The electrocardiogram (ECG) signal, in a non-invasive method, incorporates of the maternal ECG (MECG) signal, the fetal ECG (FECG) signal, and several sources of interference, such as power line interference, baseline wander, motion artifact, fetal brain activity, muscle artifact, as well as noise, such as instrumentation noise [1,2,3]. FECG signal is used to monitor the health status of the fetus by determining its maturity level, reactivity, development, and existence of fetal distress [4].

FECG extraction and enhancement method requires the elimination of the MECG as well as optimal detection of the FECG. The frequencies of both signals are a few Hertz’s and are possibly overlapping. Thus, separating them using the conventional linear filter fails. To address this problem, a large number of FECG extraction algorithms have been proposed over the past few decades. Some of these algorithms were based on the blind source separation (BSS) or blind source extraction (BSE) techniques [5,6]. In general, the extraction algorithms can be classified as either spatial (non adaptive) or temporal (adaptive) algorithms [7]. Examples of the BSS/BSE based non-adaptive algorithms include principal component analysis (PCA) [8], independent component analysis (ICA) [8,9], time scale image (TSI) and singular value decomposition (SVD)/ICA [10], periodic component analysis [11], parallel linear predictor (PLP) filters [12,13], template subtraction (TS) [14], artificial neural network (ANN) [15], support vector regression (SVR) [16], tensor decomposition (TD) [17], deflation [18], adaptive comb filter (ACF) [19], and null space component (NCA) [20]. Examples of the adaptive algorithms include the multi-sensory adaptive noise canceller (MSANC) [7], fast adaptive orthogonal group ICA [21], adaptive Volterra filter (AVF) [22], adaptive neuro fuzzy inference system (ANFIS) and wavelet transform [23], Kalman filtering [24], event synchronous canceller [25], and type-2 adaptive neuro-fuzzy inference systems [26].

The PCA method is a standard statistical technique focused on finding a transformation matrix that transforms the input data to another set of data that are uncorrelated, yielding an estimate of the unknown source signals. The method is highly affected by noise and the extraction is weak if the set of input data are statistically independent [8]. The quality of the separation was improved by employing ICA that eliminates the higher-order dependence, rather than imposing second order dependence in PCA [8]. The ICA is affected by significant noise, like PCA, and it has some limitations when used alone, unless it is combined with another method. For instance, the work in [9] used ICA to separate FECG signals and detect and classify of mother and fetal heart beats based on compressive sensing (CS) theory. ICA has been also used together with the wavelet decomposition [27], TSI and SVD [10], and adaptive noise cancellation [28], to extract FECG signals.

The work in [11] was intended to remove the MECG artifacts from FECG recording, by extracting the most periodic linear mixtures of a recorded ECG. Some extraction merits were recorded using this method. In [12], the work presented a novel BSE algorithm using a class of PLP filters whose input is the covariance matrix of the whitened data, while the estimated source signals being considered as the parallel filter coefficients. The method has the merits of solving the power level ambiguity, and has a fast convergence. The work in [13] employed the BSE based PLP to extract FFEC signals from ECG recording. The template subtraction technique was applied in [14] to remove MECG from abdominal signals using an approximation of the current MECG segment based on a linear combination of previous MECG segments aligned on the R-peak.

The work in [15] analyzed the ECG signal based on three different ANN classifiers and combined based (discrete wavelet transform and morphology) features. In [16], the SVR technique was applied to approximate the nonlinear mapping of the MECG component from thoracic signal then extract the FECG signal by subtracting the mapped thoracic signal from an abdominal signal. The technique could obtain good results for the small sample training set. The work in [17] employed a robust tensor decomposition and extended Kalman filter (EKF) to extract the FECG signal and estimate its R-peaks locations. The results showed efficient estimation of the R peaks. In [18], an online version of an iterative subspace denoising procedure was proposed for removing MECG from abdominal signals.

BSS-based ACF was presented in [19] to estimate quasi-periodic component from physiologic signal, such as ECG, by adjusting the temporal variations in fundamental frequency. The method has large FECG extraction failure and can obtain reasonable estimates of the FECG signals for approximately 60% of the abdominal signals in the database used in the paper.

The comparison between the relative performances of these algorithms is a challenging task due to the absence of a large public database and of also the absence of a defined evaluation methodology. However, it is possible to highlight the strengths and weaknesses of limited algorithms, evaluated on the same database and using the same methodology [29].

The NCA was proposed in 2007 by R. B. Chena and Y. NianWub [30] to solve the over-complete BSS problem. The solution space of the source signals were characterized by the null space of the mixing matrix using SVD. The problems were formulated in the framework of the Bayesian latent variable model. The work was only applied to three sound signals. There is no information about the performance of this approach when the number of signals is increased. The computational complexity (CC) of this algorithm was not provided. In addition, there were no comparisons with other methods. Another NCA algorithm was presented in [31] for noisy mixture. This algorithm used a transformation matrix to resolve the rotation ambiguity and extract the source signals that were assumed to be linearly independent. The initial guess of this algorithm depends more heavily on the solutions as compared with ICA. In addition, it has higher complexity than many existing ICA methods. The work in [20] presented an extension of NCA framework, named the constraint NCA (c-NCA) approach. This approach was considered as an alternative approach to the c-ICA. The c-NCA used signal-dependent semidefinite operators, which is a bilinear mapping, as signatures for operator design. A prior knowledge of how the data are prepared, collected, and mixed, is needed in this approach. This method has many issues. First, the algorithm requires a little knowledge about the sources during initialization such as imposing sparsity constraints on representing source signals [20]. This is not suitable for real-life cases. Second, the condition for convergence requires the calculation of maximal eigenvalues of the Hessian matrix of the objective function. The calculation of eigenvalues is numerically intractable. Third, the complexity of the algorithm is high and approaches $O(N_{1}N_{2}N^{3})$, where $N_{1}$ is the number of iterations, $N_{2}$ is the number of proximal splitting iterations, and *N* is the number of samples. Thus, designing new null space separating operator with less computational complexity, with no initialization constraint, and fast convergence, is crucial.

In our previous work [32], we proposed a new null space algorithm for complete and over-complete BSS of auto-regressive source signals. Matrix factorization was used to construct the separation (also called transformation) matrix. The algorithm was tested and showed successful extraction for speech and Gaussian signals. The algorithm has high computational complexity. However, it is less than that in [20]. An alternative approach of estimating the null space separation matrix with less computational complexity is possible by computing the idempotent transformation matrix (ITM) [33].

This paper is aimed to develop a non-adaptive FECG detection and extraction algorithm, based on using the null space approach in estimating the FECG and MECG signals from the ITM. The algorithm first reduces the effect of noise and interference using pre-processing filters, computes the ITM, then extracts the FECG and MECG signals from the null space of ITM. The algorithm detects also the fetal heart rate and uses it to improve the quality of extracting the FECG signal. A comparison between the proposed algorithms and other similar algorithms will be provided.

The rest of this paper is organized as follows. In Section 2, we briefly define the BSS problem and how it can be used in FECG extraction. The ECG signal is also illustrated in this section. A review on the popular FECG extraction methods (PCA, FastICA, and PLP), in the context of BSS, is shown in Section 3. In Section 4, we present the proposed FECG and MECG extraction algorithms, and how to detect the R peaks in the QRS complex. The experimental results are demonstrated and discussed in Section 5 and Section 6, respectively, as well as some topics for future work. Finally, Section 7 concludes the paper.

## 2. Problem Formulation

The biological ECG signal of a pregnant woman is a composite signal between the FECG, MECG, and the noise. It has been proven that the noiseless ECG signals can be modelled using the linear BSS model expressed by [8]:

(1) X=AS,

where X is the M×N zero mean recorded ECG mixture signals, from the thorax and the abdominal channels, A is the M×L unknown full rank mixing matrix, S is the L×N unknown source signals (the FECG and the MECG signals), recalling that *M* is the number of recorded ECG signals, *L* is the number of the unknown source signals (L≤M), and *N* is the number of samples of each measurement. We assume that both *M* and *L* are less than *N*. The matrices X and S have *M* and *L* row vector signals, respectively. Typical ECG signal is composed of P wave, QRS complex, S wave, and T wave. Both FECG and MECG signals are quasi-periodic. However, the amplitude and duration of P, QRS, and T waves are different. In addition, the FECG signal has higher frequency than the MECG signal [2,4]. The ECG signal is captured by appropriate electrodes placed at the abdominal and thorax.

The estimation of S and A from X is the main goal of the BSS problem. To estimate S, we denote matrix Y, having the same dimension of S, as the estimated source matrix, given by

(2) Y=HX,

where H is the L×M estimated transformation matrix.

As the BSS model shown in (1) is affected by scaling, permutation, and rotation ambiguities [34], several methods has been developed to extract S using (2). This will be discussed in Section 3.

## 3. Related FECG Extraction Methods

In this section, we discus some widely used approaches, such as PCA and FastICA [35,36]. Moreover, the PLP method in [12] is also included as a recent method to compare with.

### 3.1. PCA Approach

Different methods were reported in the literature to estimate H based on PCA [37]. The PCA whitening method is one of the popular methods, in which the matrix H is computed from the whitening matrix $C_{x}^{-\frac{1}{2}}$, where $C_{x}$ is the M×M covariance matrix of X. The PCA output signals have the property of being uncorrelated. However, these signals do not necessarily represent independent sources [37]. Thus, the PCA method has inferior extraction performance compared with other methods. Despite this disadvantage, the PCA algorithm has less amount of computations as compared with other methods, and shows acceptable detection of FECG R peaks. Thus, the PCA method is still showing interest by researchers in the field of FECG detection and extraction [36,38,39].

### 3.2. FastICA Approach

In the FastICA approach, the matrix H is equal to $A^{+}$, which is the Moore–Penrose inverse of A, such that $A^{+}=A^{T}{\left({AA}^{T}\right)}^{-1}$, if L≤M [12]. The resultant estimated sources must be statistically independent. In many FastICA algorithms, the whitening process is needed prior to applying the algorithm, to minimize the correlation between the mixture signals. The PCA approach may be used for data whitening. The FastICA based FECG extraction has some challenges. First, it assumes independent sources and its performance is directly affecting the quality and speed of FECG signal extraction. Second, the background noise has a considerable affect on its performance [3]. Some works were reported to combine the FastICA approach with other approaches [9,10,27,28].

### 3.3. BSE Based PLP Filter

In [12], a BSE based PLP filter was proposed to solve BSS problem using a set of linear predictors that works simultaneously to predict the unknown input sources. The input to the PLP filter is the covariance matrix of the whitened data, denoted by R(n), with the estimated source signals being considered as the PLP filter coefficients. The number of coefficients is fixed to *N* in this method. The prediction error is minimised using the standard gradient descent algorithm (GDA). The update equation of the estimated source signals, is given by:

(3) $$y_{m}\left(n+1\right)=y_{m}\left(n\right)+\mu_{y}\left(R\left(n\right)-I_{N}\right)E_{r}\left(n\right),$$

where $y_{m}\left(n\right)={\left[y_{m}\left(1\right),y_{m}\left(2\right),\ldots ,y_{m}\left(N\right)\right]}^{T}$, is the $m^{th}$ vector, m = 1, 2, …, L, of the estimated source signal, $y_{m}\left(n+1\right)$ is the update vector of $y_{m}\left(n\right)$, $\mu_{y}$ is the learning rate, and $E_{r}\left(n\right)$ is the error vector. The method has the merits of solving the mixture power ambiguity and has a fast convergence rate. The method was employed to extract FECG signal from real and synthetic ECG data [13]. In addition, the method can be used to extract signal from a noisy mixture.

## 4. The Proposed FECG Extraction System

The proposed system is based on extracting the FECG and MECG signals from the null space solution of a certain matrix, named idempotent transformation matrix (ITM), and is being referred to us as W. In this section, we first discus the pre-processing stage that is required to reduce the effect of noise and interface in the ECG signals, since real ECG signals are usually contaminated with different types of noise and interference, as declared in Section 1. Then, the derivation of W matrix is provided. A solution method, using the null space of W, is then explained to address the extracted FECG and MECG signals. We named the extracted FECG signal as raw FECG since it may be contaminated with some unwanted MECG peaks. Thus, a post-processing stage is required to clean the raw FECG signal from the unwanted MECG peaks.

### 4.1. The Pre-Processing Stage

The pre-processing stage includes three cascaded stages: low pass, high pass, and notch filters. The input and output of the the preprocessing stage are defined by X and $X_{p}$, respectively. A low pass Butterworth filter with 100 Hz cutoff frequency, defined by $f_{1}$, is applied to limit the frequency band of the input ECG signals. The baseline wander effect is reduced using high pass filter of 0.5 Hz cutoff frequency, defined by $f_{2}$, [29]. A second order notch filter having cutoff frequency, defined by $f_{3}$, of 50 Hz or 60 Hz is used to remove the 50 Hz or the 60 Hz power line interference, respectively. The selection of the cutoff frequency depends upon the power line standard which is either European or US standard [9].

### 4.2. The Idempotent Transformation Matrix (ITM)

Define the *j*th signal $y_{j}\left(n-k+1\right)$, j=1,2,…,L, k=1,2,…,N, as the extracted FECG or MECG source signal, and expressed by the following *N* prediction filter [32,33,40]

(4) $$y_{j}\left(n-k+1\right)=\sum_{q=1}^{N}w_{q,k}y_{j}\left(n-q+1\right),$$

where $w_{q,k}$ is the autoregressive coefficients of $y_{j}\left(n-k+1\right)$. Re-writing (4) in matrix form, we obtain

(5) Y=YW,

where Y is the extracted source matrix of dimension L×N, and can be written as

(6) $$Y=\left[\begin{array}{cccc} y_{1}\left(n\right) & y_{1}\left(n-1\right) & \ldots & y_{1}\left(n-N+1\right)\\ y_{2}\left(n\right) & y_{2}\left(n-1\right) & \ldots & y_{2}\left(n-N+1\right)\\ \vdots & \vdots & \vdots & \vdots \\ y_{L}\left(n\right) & y_{L}\left(n-1\right) & \ldots & y_{L}\left(n-N+1\right) \end{array}\right],$$

and W is the N×N symmetrical idempotent transformation matrix, and is given by

(7) $$W=\left[\begin{array}{cccc} w_{1,1} & w_{1,2} & \ldots & w_{1,N}\\ w_{2,1} & w_{2,2} & \ldots & w_{2,N}\\ \vdots & \vdots & \vdots & \vdots \\ w_{N,1} & w_{N,2} & \ldots & w_{N,N} \end{array}\right].$$

The matrix W can be computed as follows [33]:

(8) $$W=\frac{1}{N}X_{p}^{T}C_{x}^{-1}X_{p},$$

where $C_{x}$ is the covariance matrix of X. The computational complexity of W is equal to $O(M^{4}N^{2}+6N^{3}+\frac{14}{3}N^{3})$. However, it can be reduced to $O\left(M^{2}N^{2}+MN^{2}\right)$ using an iterative method [33].

### 4.3. The Null Space Solution of W

The matrix W, computed from Section 4.2, is then used to estimate the extracted raw FECG and MECG signals, as follows:

Equation (5) can be rewritten as

(9) $$QY^{T}=0_{N\times L},$$

(10) $$Q=W-I_{N},$$

where Q is the required N×N separation matrix, and $0_{N\times L}$ is an N×L zero matrix. Equation (9) can be solved for the unknown Y using the null space of Q, as follows:

(11) Y=Null(Q).

**Figure 1:**
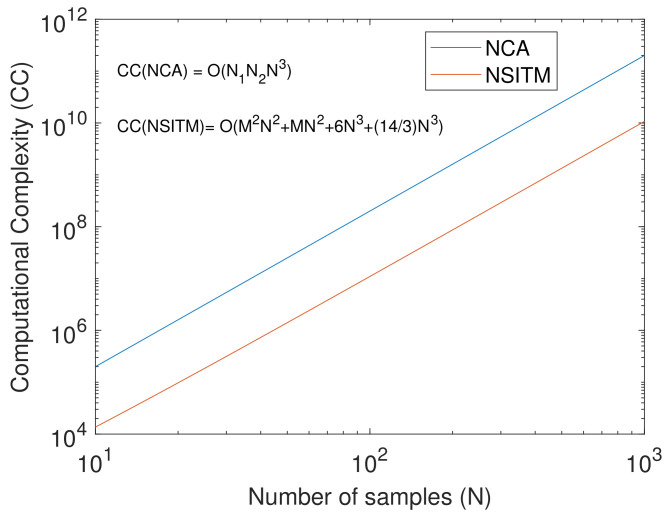
A logarithmic plot to illustrate a comparison between the computational complexity (CC) of NCA and NSITM, with the variations of number of samples (N), assuming $N_{1}=20$, $N_{2}=10$, and M=5.

Since the extracted signals of the Y matrix are computed based on (11), i.e, the Null space (NS) of Q, and since W is an ITM, we call this method NSITM. The computational complexity of Null(.) based on SVD is equal to $O(6N^{3}+\frac{14}{3}N^{3})$ [41]. Thus, the overall computational complexity of NSITM will be $O\left(M^{2}N^{2}+MN^{2}+\frac{14}{3}N^{3}\right)$. It is clear that there is a significant reduction in the computational complexity of the proposed NSITM as compared with the NCA method in [20]. Figure 1 shows a logarithmic plot to illustrate a comparison between the computational complexity of NCA and NSITM when *N* is varied from 10 to 1000, assuming $N_{1}=20$, $N_{2}=10$, and M=5.

The solution of (11) can be obtained using SVD. First, we express Q by

(12) $$Q=U_{q}D_{q}V_{q}^{T},$$

where $U_{q}$ is an N×N unitary matrix, $D_{q}$ is an N×N diagonal matrix with the eigenvalues of Q, and $V_{q}$ is an N×N matrix with the columns being the eigenvectors of Q. Assume that $V_{q}$ is expressed by

(13) $$V_{q}=\left[\begin{array}{cccc} v_{1,1} & v_{1,2} & \ldots & v_{1,N}\\ v_{2,1} & v_{2,2} & \ldots & v_{2,N}\\ \vdots & \vdots & \vdots & \vdots \\ v_{N,1} & v_{N,2} & \ldots & v_{N,N} \end{array}\right],$$

then, from (11)–(13), and since L<M, which is the usual case in FECG extraction, the solution Y will be taken from the last *L* column vectors of $V_{q}$, and is given by

(14) $$Y={\left[\begin{array}{cccc} v_{1,N-L+1} & v_{1,N-L+2} & \ldots & v_{1,N}\\ v_{2,N-L+1} & v_{2,N-L+2} & \ldots & v_{2,N}\\ \vdots & \vdots & \vdots & \vdots \\ v_{N,N-L+1} & v_{N,N-L+2} & \ldots & v_{N,N} \end{array}\right]}^{T}.$$

Equation (14) represents the extracted raw FECG and MECG signals.

**Figure 2:**
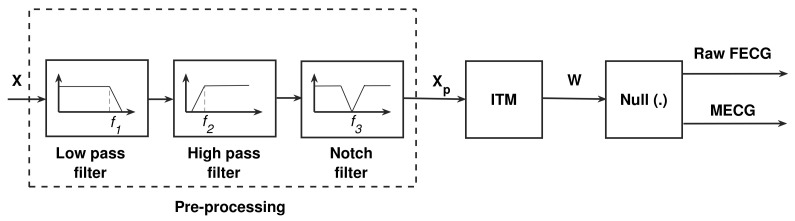
Raw FECG and MECG extraction system. The cut-off frequencies $f_{1}$, $f_{2}$, and $f_{3}$ are 100 Hz, 0.5 Hz, and 50/60 Hz.

Figure 2 illustrates the block diagram of the raw FECG and MECG system, based on the discussion in Section 4.1, Section 4.2 and Section 4.3.

As the MECG signal level, in the input mixture, is high as compared with the FECG signal, the MECG signal may exist in the extracted FECG signal, especially in noisy environments. This needs a postprocessing stage to detect first the MECG peaks and then remove them from the raw FECG signal. This post-processing is shown in Section 4.4.

### 4.4. The Post-Processing Stage

The operation of this stage is based on using multiple window functions, named adaptive comb filter (ACF) [19], centered at all MECG peaks, then multiplying its unit sample response by the raw FECG signal. This results in removing the unwanted MECG component from the raw FECG signal. The post-processing stage consists of peaks detection, control logic, and MECG removal. In the following, we will discuss all these stages and then show how to connect them in order to remove the unwanted MECG peaks from the raw FECG signal.

#### 4.4.1. Peaks Detection

The R peaks in the raw FECG and MECG QRS complex are detected using the Pan–Tompkins algorithm [42,43]. Due to the quasi-periodic nature of the FECG and MECG signals, and since the time needed to record ECG signals is typically long and contains many periods of the signals, we define $p_{1}$ and $p_{2}$ as vectors that contain the sampling indices, i.e., locations, of all detected MECG and FECG peaks, respectively. We also define $P_{1}$ and $P_{2}$ as the number of detected MECG and FECG peaks, respectively. Then, the difference in sampling indices between two consecutive MECG and FECG peaks, defined as $dp_{1}$ and $dp_{2}$, are given by

(15) $$dp_{1}\left(l\right)=p_{1}\left(l+1\right)-p_{1}\left(l\right),\;\;\;l=1,2,\ldots ,P_{1}-1,$$

(16) $$dp_{2}\left(k\right)=p_{2}\left(k+1\right)-p_{2}\left(k\right),\;\;\;k=1,2,\ldots ,P_{2}-1.$$

The *l*th maternal heart rate (MHR*_l_*) and the *k*th fetal heart rate (FHR*_k_*) can be calculated from (15) and (16), as follows:

(17) $$MHR_{l}=60f_{s}/dp_{1}\left(l\right),$$

(18) $$FHR_{k}=60f_{s}/dp_{2}\left(k\right),$$

where $f_{s}$ is the sampling frequency of the ECG signals. The average values (MHR and FHR) are then calculated by

(19) $$MHR=\frac{1}{P_{1}-1}\sum_{l=1}^{P_{1}-1}MHR_{l},$$

(20) $$FHR=\frac{1}{P_{2}-1}\sum_{k=1}^{P_{2}-1}FHR_{k}.$$

#### 4.4.2. Control Logic

The control logic stage is required to decide about the existence of unwanted MECG peaks at the raw FECG signal, then activate the MECG removal stage when needed. The stage checks the estimated FHR. If the raw FECG signal contains MECG components, then the estimated FHR from (20) will not fall within the expected FHR ranges, since the values of elements of the vector $dp_{2}$ will decrease according to (16), resulting in an increase in FHR*_k_* according to (18). For instance, if an MECG peak is located half the way between two consecutive FECG peaks, the local values of the elements of $dp_{2}$ in that sector will be decreased to half its value as compared with the situation when MECG peak does not exit. This causes the FHR*_k_* to be doubled its value in that sector. If an MECG peak is very close to the location of the FECG peak, then $dp_{2}$ local value approaches zero in that sector, resulting in an infinite value of FHR*_k_*. As a result, the existence of MECG peaks causes a large increase in the variance of the estimated vector [FHR_1_, FHR_2_, …, FHR_*P*_2_−1_]. The control logic considers these two factors and activates the MECG removal to extract a clean FECG signal. However, if the MECG peaks do not exist in the raw FECG signal, the control unit will deactivate the MECG removal stage. Thus, the raw FECG signal will be considered as the clean FECG signal.

#### 4.4.3. MECG Removal

The MECG removal stage removes the MECG components in the raw FECG signal using ACF, whose unit sample response is denoted by h(n), and is expressed as

(21) $$h(n)=\sum_{q=1}^{P_{1}}w\left(n-p_{1}\left(q\right)\right),$$

(22) $$w(n)=\sum_{r=-U}^{U}a_{r}\delta \left(n-r\right),$$

(23) $$a_{r}=0.46-0.46cos(\frac{2\pi r}{2U+1}),$$

where w(n) is the unit sample response of a non-causal Hamming window with 2U+1 being its length, and centered at n=0, $a_{r}$ is the window coefficient, and δ(n) is a unit sample function. The resultant h(n) consists of multiple window functions centered at all MECG peaks $p_{1}\left(1\right),p_{1}\left(2\right),\ldots ,p_{1}\left(P_{1}\right)$—thus multiplying h(n) by the raw FECG results in removing the unwanted MECG components.

**Figure 3:**
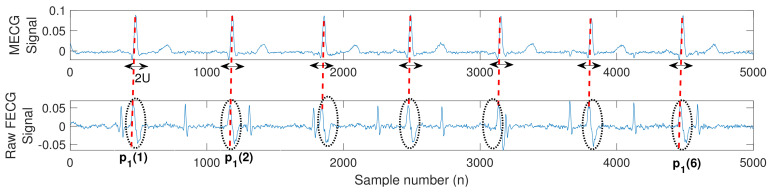
Typical extracted MECG and raw FECG signals from the system shown in Figure 2. The red dashed lines and the black dotted circles represent the locations ($p_{1}\left(1\right),p_{1}\left(2\right),\ldots ,p_{1}\left(6\right)$) and the components of the unwanted MECG signals in the raw FECG signal, respectively. The doubled black arrows indicate the locations of the adaptive comb filter (ACF), of length 2U+1, required to remove the MECG components.

Figure 3 illustrates an example of a typical extracted MECG and raw FECG signals using the system shown in Figure 2. The figure illustrates how to position the ACF in order to remove the unwanted MECG components from the raw FECG signal. More details about MECG removal are provided in the simulation section.

**Figure 4:**
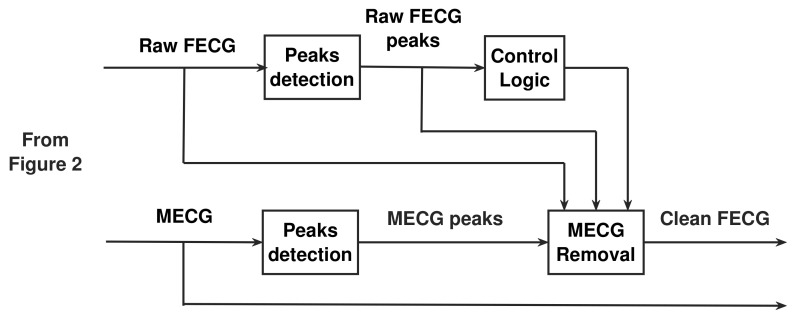
The proposed post-processing stage used to get the clean FECG signal.

**Figure 5:**
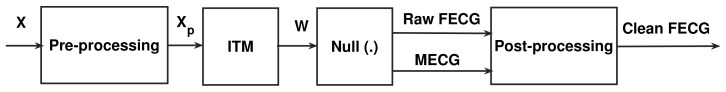
The proposed NSITM system.

Figure 4 illustrates the proposed post-processing stage used to get the clean FECG signal. Figure 5 illustrates the proposed NSITM system based on all discussions in Section 4.1, Section 4.2, Section 4.3 and Section 4.4.

### 4.5. The Proposed NSITM Algorithm

From Figure 5, we propose the NSITM Algorithm 1 that extracts the MECG/FECG signals from the ECG mixture signals. The maxFHR is the maximum possible FHR. At 20 weeks, it can be set to 180 beats per minute (bpm) [2]. The maxvar is the maximum variance of FHR vector [FHR_1_, FHR_2_, … FHR_*P*_2_−1_]. Values of 1–5 are found to be appropriate during simulation. The var(.) used in the algorithm represents the variance of (.).
**Algorithm 1** The proposed NSITM extraction algorithm.1:**Initials***N*, *L*, *M*, maxFHR, maxvar.2:Read the ECG signals X.3:Preprocessing by denoising filters (low pass, high pass, notch).4:Compute W by (8).5:Compute Q by (10).6:Compute $V_{q}$ by (12) using SVD method.7:Compute Y by (14).8:Compute MECG peaks locations by [42,43].9:Compute MHR by (19).10:Compute raw FECG peaks locations by [42,43].11:Compute FHR by (20).12:V = [FHR_1_, FHR_2_, …, FHR_*P*_2−1__].13:**if**(var(V)>maxvar and FHR >maxFHR)14: Compute h(n) by (21)–(23).15: Remove MECG component by: Clean FECG = h(n). Raw FECG.16:**else**17: Clean FECG = Raw FECG.18:**endif**19:**Return** clean FECG, MECG.

## 5. Experiments

Five different simulations are provided in this section. The first simulation uses real ECG signals from the database for the Identification of Systems (DAISY) [44]. Then, the FECG signal is extracted using our proposed NSITM algorithm. The simulation is repeated using PCA, FastICA, and PLP algorithms, for comparison purposes. The second simulation is similar to the first simulation but uses another real piece of data from Physionet/Computing in the Cardiology Challenge 2013 database [45,46]. The third simulation extracts the FECG signals from a synthesized ECG data and then evaluates their performances. The synthesized data were taken from Physionet/Fetal ECG Synthetic database (FECGSYNDB) [46,47]. The fourth simulation investigates the FECG extraction metrics based on fetal-to-maternal SNR (fmSNR) variations. Data used in this simulation are the same as the data used in experiment 3. The fifth simulation evaluates the performance of the algorithm using statistical measures, using the data from experiment 2. In all simulations, the denoising filters (lowpass, highpass, and notch) were performed in all algorithms used in this work. In addition, for clarity, all signals are visualized in a normalized form, unless otherwise specified. All simulations were conducted in Matlab R2018b, on 2.2 GHz Intel Core i7-8750 CPU with 16 GB RAM, Windows 10.

### 5.1. Experiment 1: FECG Extraction of Real ECG Data from the DAISY Database

Recorded real ECG signals, from pregnant women for 10 s, were used from [44]. The signals were acquired from eight channel sensors (five abdominal and three thorax channels). The sampling frequency $f_{s}$ was selected to be 250 Hz. Then, the proposed NSITM algorithm was applied to extract the FECG and MECG signals. The PCA, FastICA, and PLP algorithms were also applied to extract the FECG and MECG signals, and their results will be compared with the results from the proposed algorithm.

**Figure 6:**
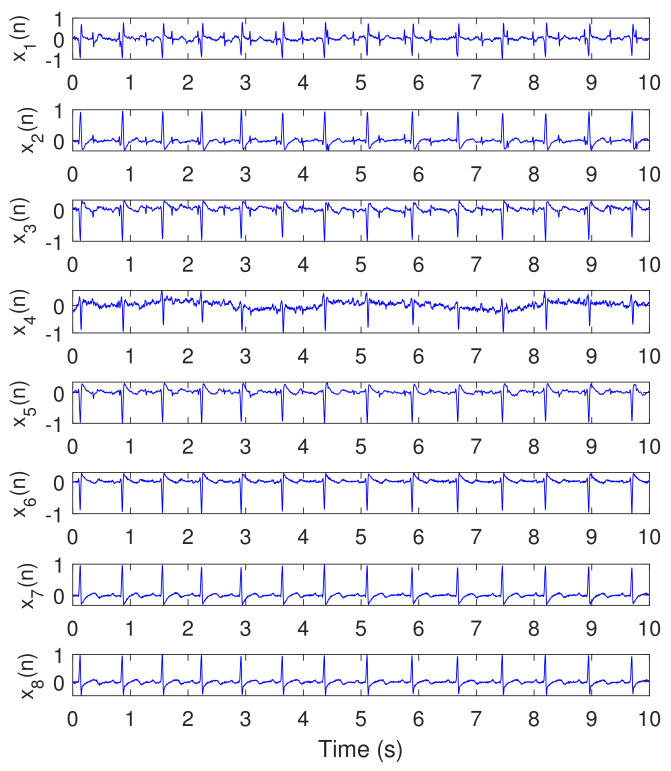
Recorded ECG signals using DAISY data set, N=2500, M=8, $f_{s}=250$ Hz. The abdominal signals are the first five signals from the top ($x_{1}\left(n\right)$–$x_{5}\left(n\right))$ while the remaining three ($x_{6}\left(n\right)$–$x_{8}\left(n\right))$ are thorax signals.

**Figure 7:**
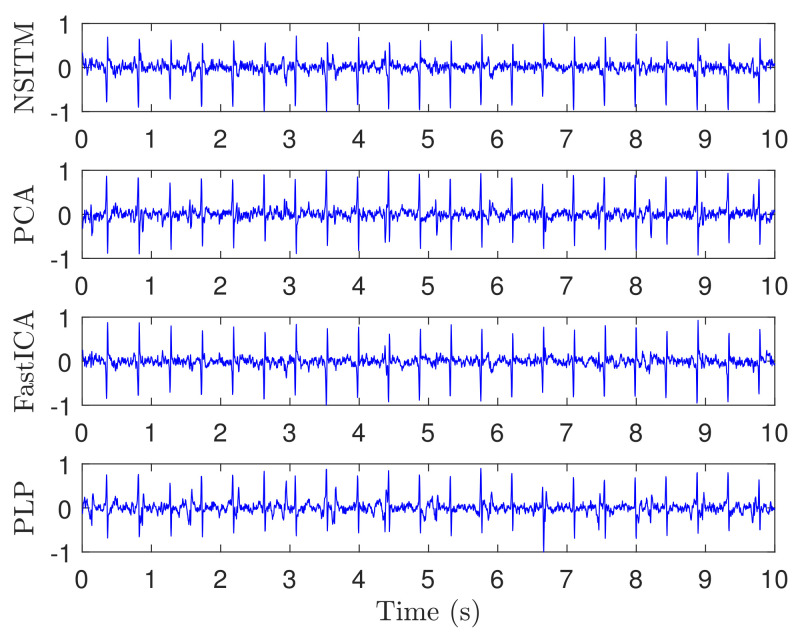
Extracted FECG signals from ECG signals in Figure 6, using NSITM, PCA, FastICA, and PLP, assuming M=7 (five abdominal signals $x_{1}\left(n\right)$–$x_{5}\left(n\right)$ and two thorax signals $x_{6}\left(n\right)$–$x_{7}\left(n\right)$.The *y*-axis labels refer to the names of the algorithms used for extracting the FECG signals. Data used are from the DAISY data set.

**Figure 8:**
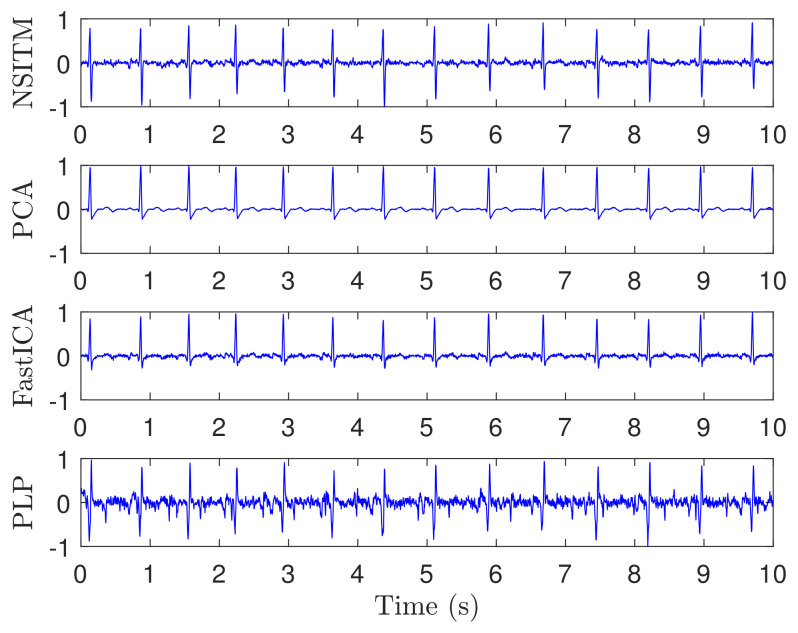
Extracted MECG signals from ECG signals in Figure 6, using NSITM, PCA, FastICA, and PLP, assuming M=7 (five abdominal signals $x_{1}\left(n\right)$–$x_{5}\left(n\right)$ and two thorax signals $x_{6}\left(n\right)$–$x_{7}\left(n\right)$. The *y*-axis labels refer to the names of the algorithms used for extracting the MECG signals. Data used are from the DAISY data set.

Figure 6 illustrates the recorded ECG signals, with N=2500 samples, and M=8, using five abdominal signals ($x_{1}\left(n\right)$–$x_{5}\left(n\right)$) and three thorax signals ($x_{6}\left(n\right)$–$x_{8}\left(n\right)$). There are a large number of combinations between these eight signals needed as input to test the algorithms. In our work, and to efficiently use the space, we showed only the case for (M=7). Figure 7 and Figure 8 illustrate the extracted FECG and MECG signals, respectively, using the selected algorithms, considering five abdominal signals ($x_{1}\left(n\right)$–$x_{5}\left(n\right)$) and two thorax signals ($x_{6}\left(n\right)$–$x_{7}\left(n\right))$. As the data from DAISY are clean, the MECG components were not found in the raw FECG signals, thus the control unit in Figure 2 will deactivate the MECG removal stage. Thus, the extracted FECG signal shown in Figure 7 represents both the raw FECG and the clean FECG signals. The visualization on the results indicates that the proposed NSITM algorithm, the PCA algorithm, the FastICA, and PLP algorithms are effective in extracting the FECG and MECG signals from the ECG mixture. In addition, the estimated FECG signals using NSITM and FastICA show less noise contents as compared with PCA and PLP.

### 5.2. Experiment 2: FECG Extraction of Real ECG Data from the Physionet Database

Recorded real ECG signals, from pregnant women for one minute, were used from the Physionet Challenge 2013 data set [45,46]. Each recording includes four noninvasive abdominal signals. The data were obtained from multiple sources using a variety of instrumentation with differing frequency response, resolution, and configuration. The sampling frequency $f_{s}$ for all data are 1 kHz. We selected the data files (a04, a08, a14, a15, a25) from the database, and used them in this experiment. Then, we followed the same simulation procedure as in Section 5.1. For illustration purposes, we visualize only the results of file a15 due to the excessive number of figures.

**Figure 9:**
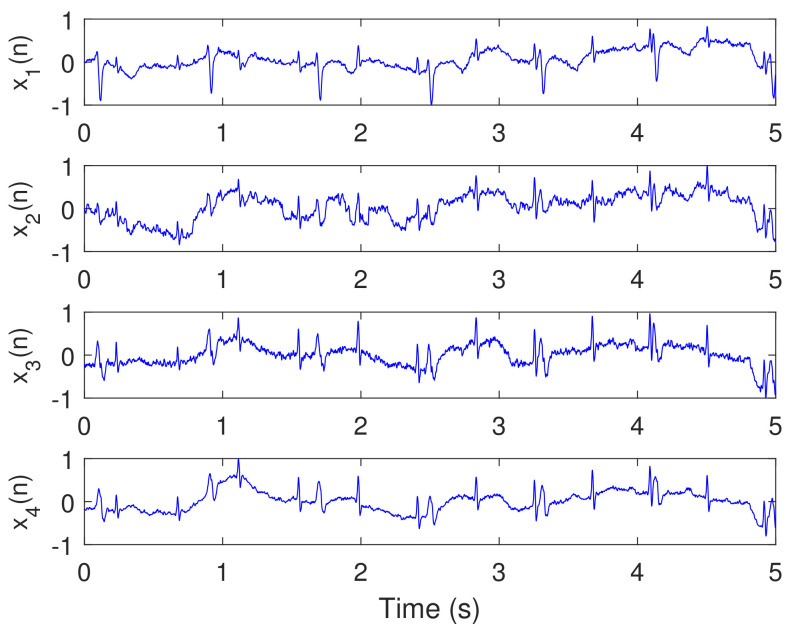
Recorded abdominal ECG signals $x_{1}\left(n\right)$–$x_{4}\left(n\right)$ from the Physionet Challenge 2013 data set a, file a15, M=4, N=5000, $f_{s}=1$ kHz, and data samples from 0–4999.

**Figure 10:**
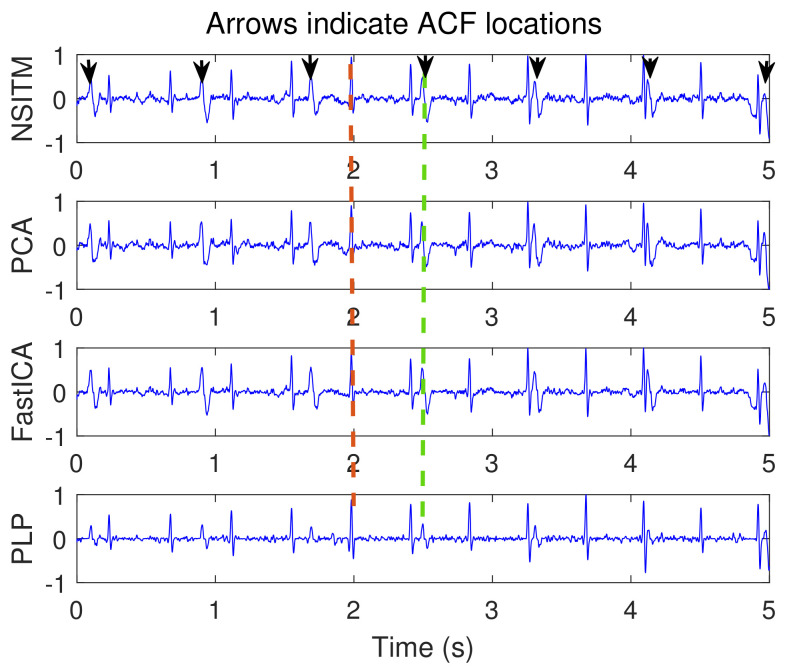
Raw FECG signals from ECG signals in Figure 9, using NSITM, PCA, FastICA, and PLP, assuming M=4. Both FECG and MECG R peaks exist. For illustration, only one marked FECG peak and one marked MECG peak are shown by red dashed lines (left located) and green dashed lines (right located), respectively. The black arrows indicate the position of the ACF used to remove the MECG R peaks.The *y*-axis labels refer to the names of the algorithms used for extracting the FECG signals. The data used are from the Physionet Challenge 2013 data set a, file a15.

**Figure 11:**
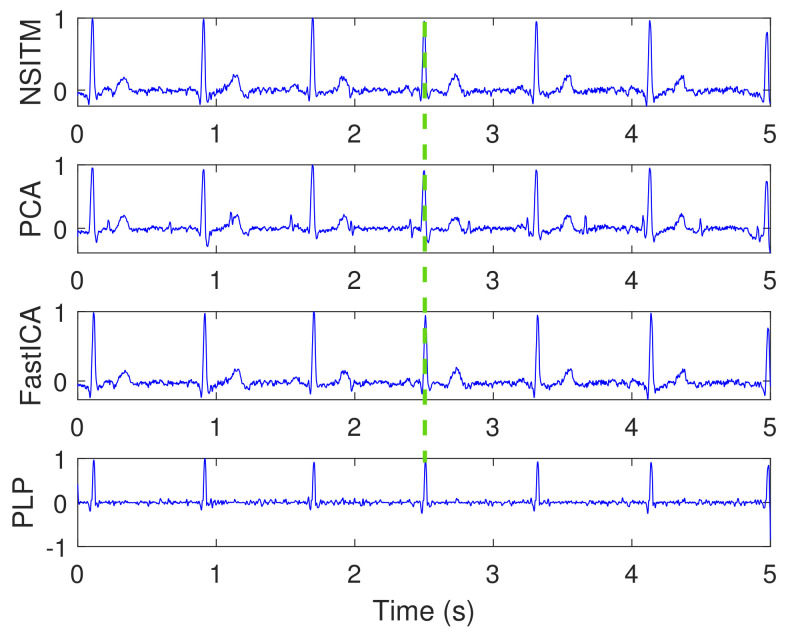
Extracted MECG signals from ECG signals in Figure 9, using NSITM, PCA, and FastICA, and PLP. Only MECG R peaks exist. For illustration, only one marked MECG peak is shown by green dashed lines. The *y*-axis labels refer to the names of the algorithms used for extracting the MECG signals. The data used are from the Physionet Challenge 2013 data set a, file a15.

Figure 9 illustrates the recorded abdominal ECG signals, from file a15, with M=4. We selected a block of 5000 data samples, from 0–4999. Figure 10 and Figure 11 illustrate the extracted FECG and MECG signals, respectively, using the selected algorithms. Figure 10 shows the raw FECG signals using all algorithms (the proposed NSITM algorithm, as well as the PCA, FastICA, and PLP algorithms). However, all used algorithms are effective in extracting the MECG signals from the ECG mixture, as in Figure 11. Figure 10 shows that both FECG and MECG R peaks exist, and are marked by red dashed lines and green dashed lines, respectively. The red dashed lines and the green dashed lines are the left and the right lines in Figure 10, respectively. Thus, the control unit will activate the MECG removal stage. The locations of the ACF, used to remove the unwanted MECG peaks, are illustrated in Figure 10 by black arrows. The length of the ACF window is a variable quantity and depends upon the duration of the QRS complex of the selected MECG signal. In this simulation, a length of 20 samples were found appropriate in removing the MECG R peaks, for the used file a15. For other used files, the length of ACF must be selected between 20 and 45 samples, to avoid the removal of portions of the required FECG signal when the two signals are very close in their locations.

**Figure 12:**
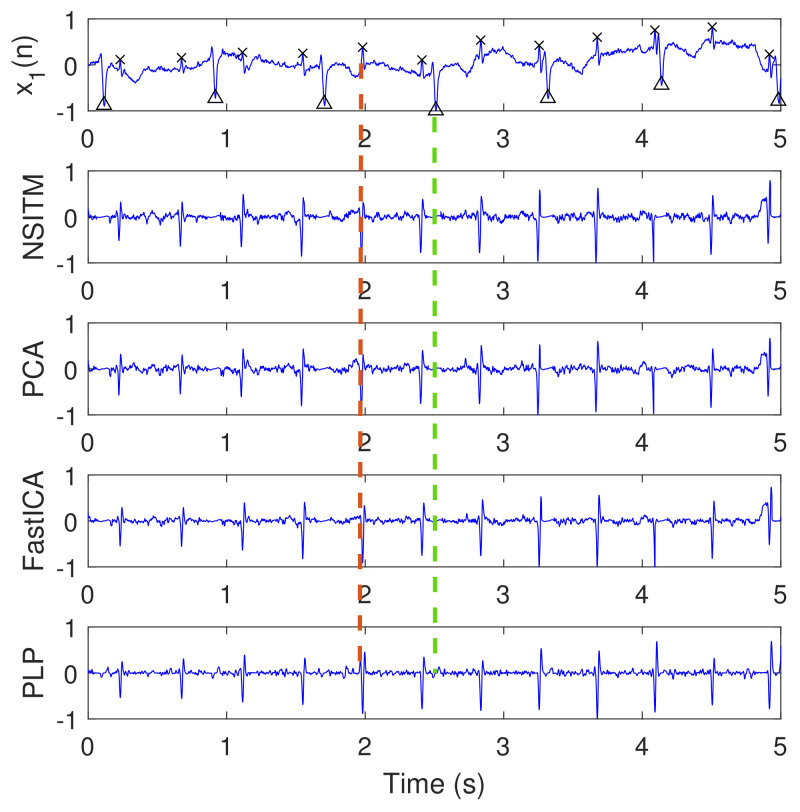
Clean extracted FECG signals (signals 2, 3, 4, and 5, from the top) of Figure 10 after the removal of MECG signals by ACF, and based on R peaks locations in Figure 11. The first signal from the top, $x_{1}\left(n\right)$, is the abdominal signal taken from Figure 9, and used as a reference of marking the FECG and MECG R peaks. The ‘x’ and ‘Δ’ markers refer to the reference positions of the R peaks in FECG and MECG signals, respectively. The red dashed lines refers to one position of the extracted FECG R peaks. The green dashed lines refers to one position of the removed MECG R peaks. The *y*-axis labels of signals 2, 3, and 4 (from the top) refer to the names of the algorithms used for extracting the FECG signals. The data used are from the Physionet Challenge 2013 data set a, file a15.

Figure 12 illustrates the clean extracted FECG signals of Figure 10 after the removal of MECG signals by ACF. The first signal from the top is the abdominal ECG signal $x_{1}\left(n\right)$, which is considered at the top of the figure for illustration purposes, since it contains the reference annotation taken from LightWAVE annotation viewer [45]. It is clear from Figure 12 that the proposed NSITM algorithm, the PCA algorithm, the FastICA algorithm, and the PLP algorithm are effective in extracting the FECG and MECG signals from the ECG mixture. The extraction performances will be considered later in Section 5.3.

To ensure the stability of extraction performance over time, the simulation is repeated by taking blocks of data samples from 5000–9999, and from 55,000–59,999 which are the last available data samples. The results obtained are very similar to the results using data samples from 0–4999. Results from those simulations are not shown in this paper to limit the number of pages. In general, the proposed NSITM algorithm as well as the other algorithms are effective in extracting both FECG and MECG signals from the abdominal ECG mixture if ACF is used to remove the MECG R peaks from the raw FECG signals shown in Figure 10.

### 5.3. Experiment 3: FECG Extraction Using Synthesized ECG Data

To study the extraction performance of the proposed algorithm, the ECG signals (FECG and MECG) must be first modelled then mixed according to (1). The modelling of ECG signals involves the generation of P, QRS, and T waves. This can be accomplished using the synthesized data taken from Physionet/Fetal ECG Synthetic database (FECGSYNDB) [46,47]. This database and its collection methods are described in [48]. Each signal had a duration of 5 minutes, and was sampled at 250 Hz with a 16-bit resolution. The FECG and MECG signals are generated by treating each abdominal signal component (e.g., foetal/maternal ECG or noise signals) as an individual source, whose signal is propagated onto the observational points, also called the electrodes. Thus, the database provides separate waveform files for each signal source [46,47]. The simulator generates 34 ECG channels (32 abdominal and 2 maternal ECG reference channels). Adding the three individual signals (FECG, MECG, and noise) per channel is then needed to generate the ECG mixture [49]. In our experiment, we consider four abdominal channels (10, 11, 18, 19) and the two reference channels (33 and 34) with different signal to noise ratio (SNR), equals to 0 dB, 3 dB, 6 dB, 9 dB, and 12 dB, respectively. The values of SNR are selected from the available values in [47]. We select eight pregnant women with simulated pregnancy numbers (01, 02, 03, 06, 07, 08, 09, 10). The selected event is maternal heart rate (MHR)/FHR acceleration/deceleration plus noise. As there are many entries needed to download a file, the file name format is long. To simplify the file format and use it in the paper, we propose a short file format. Table 1 illustrates examples of how to rename the downloaded files for different simulated pregnancy numbers, SNR, and signal type. Other file names can also be obtained based on this table.

**Figure 13:**
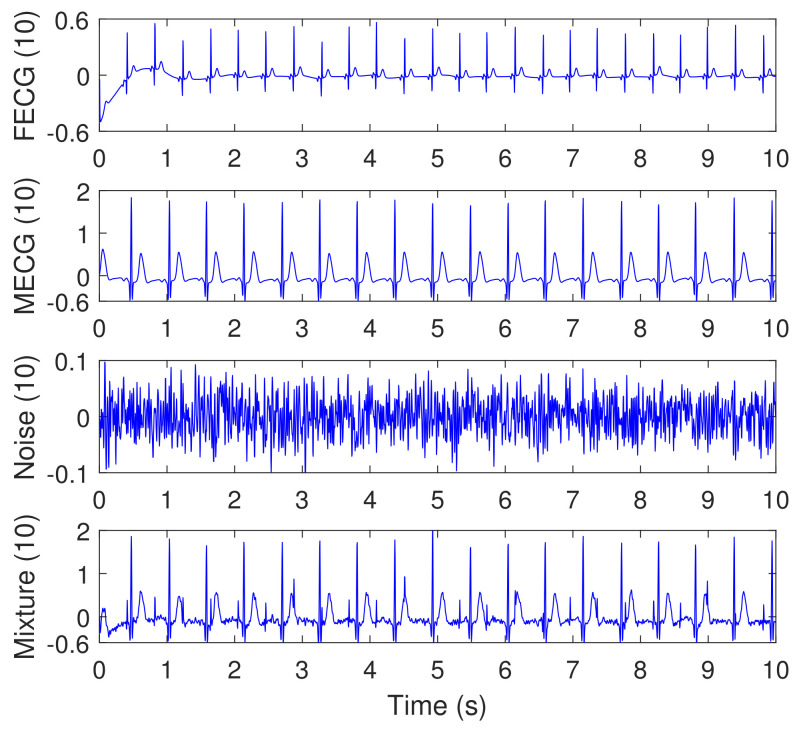
Synthesized abdominal FECG, MECG, and Noise signals (the three signals from the top), taken from channel (10), using Physionet/Fetal ECG Synthetic database (FECGSYNDB), considering simulated pregnancy number = 01, SNR = 12 dB, and event of MHR/FHR acceleration/deceleration plus noise. The corresponding paper file names are F0112, M0112, and N0112, according to Table 1. The signal number (4) from the top is the mixture signal after adding the FECG, MECG, and noise signals. The *y*-axis labels refer to the namess of signals taken from channel 10.

Figure 13 illustrates the synthesized abdominal FECG, MECG, and noise signals, from channel (10), considering simulated pregnancy number = 01, SNR = 12 dB, and event of MHR/FHR acceleration/deceleration plus noise. The signal number (4) from the top is the mixture signal after adding the FECG, MECG, and noise signals. Other signals from channels (11, 18, 19) and their corresponding mixtures were not shown in the paper due to excessive number of figures. All signals in Figure 13 are visualized in un-normalized forms to show the actual amplitudes of the components of the mixture signal (10).

**Figure 14:**
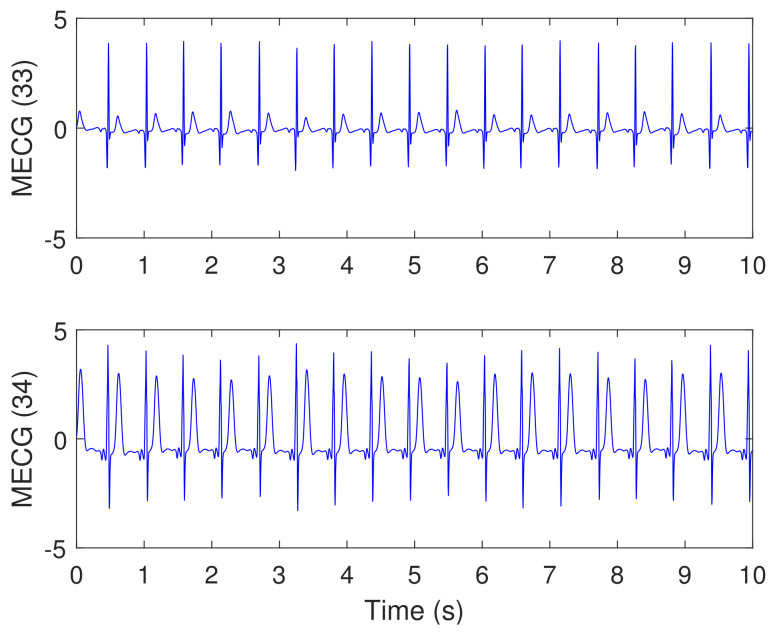
Synthesized maternal reference ECG (MECG) signals, taken from channels (33 and 34), using Physionet/Fetal ECG Synthetic database (FECGSYNDB)—assuming the same simulation settings used in Figure 13. The corresponding paper file name is M0112, according to Table 1. The *y*-axis labels refer to the names of two signals taken from channels 33 and 34.

**Figure 15:**
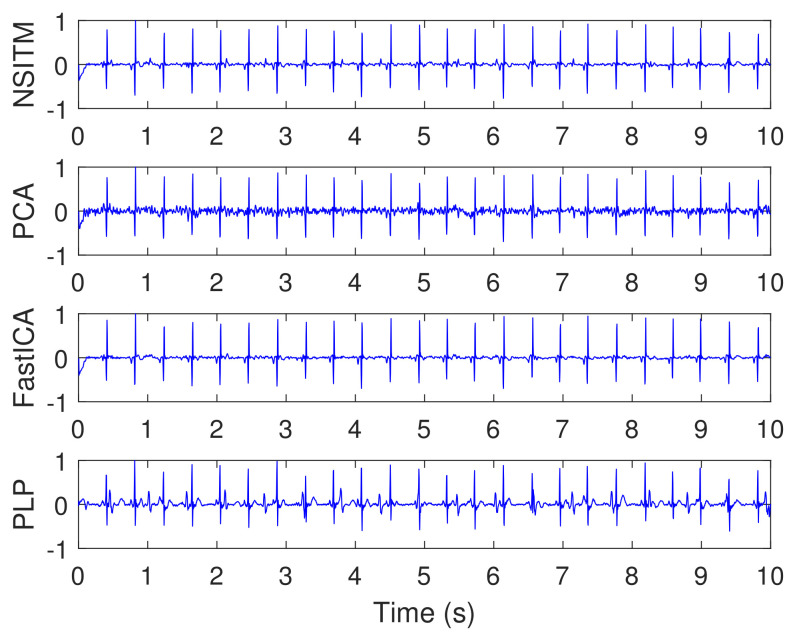
Extracted FECG signals using NSITM, PCA, and FastICA algorithms, using data shown in Figure 13 and Figure 14. The *y*-axis labels refer to the names of the algorithms used for extracting the FECG signals.

**Figure 16:**
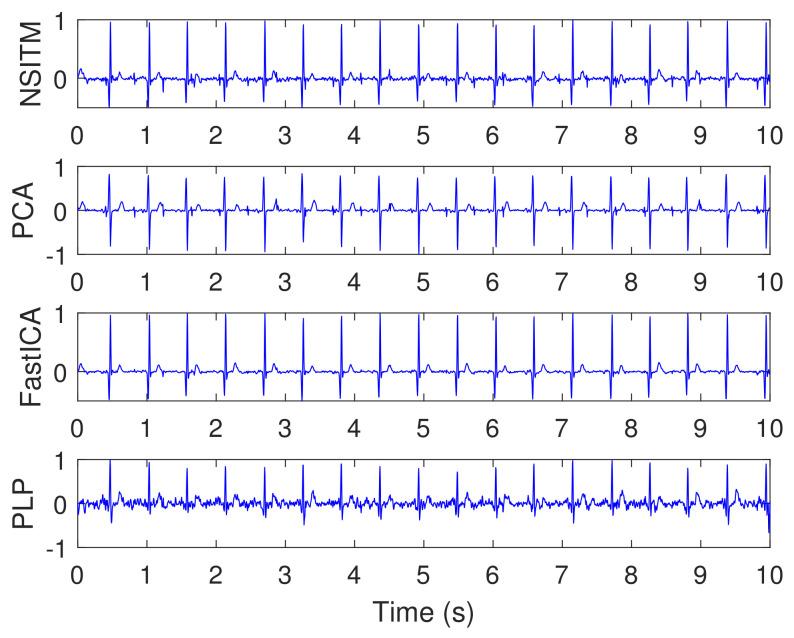
Extracted MECG signals using NSITM, PCA, and FastICA algorithms, using data shown in Figure 13 and Figure 14.The *y*-axis labels refer to the names of the algorithms used for extracting the MECG signals.

Figure 14 illustrates the synthesized maternal reference ECG (MECG) signals, from channels (33–34). All signals in Figure 14 are visualized in un-normalized forms, in order to compare it with the mixture signal (10) shown in Figure 13. The proposed NSITM algorithm was then applied to these six signals (the four abdominal mixture signals plus the two reference signals) to extract the FECG and MECG signals. The simulation is repeated to extract the FECG and MECG based on PCA, FastICA, and PLP algorithms, for comparison purposes. The extracted FECG and MECG signals from all algorithms are illustrated in Figure 15 and Figure 16, respectively. As the synthesized data are clean, the MECG components were not found in the raw FECG signals, thus the control unit in Figure 2 will deactivate the MECG removal stage. Comparing the synthesized FECG and MECG signals shown in Figure 13 with the extracted FECG and MECG signals shown in Figure 15 and Figure 16, it is clear that all algorithms are effective in extracting FECG and MECG signals from their mixture, since all extracted signals (MECG and FECG) match the original signals (MECG (10) and FECG (10)), respectively.

To evaluate the FECG extraction performance of the previous simulation, we use the similarity performance index (SPI) [7,33], the source-to-interference ratio (SIR), the source-to-artifacts ratio (SAR), and the source-to-distortion ratio (SDR) [50]. These metrics were widely used in evaluating the extraction performance of speech and biomedical signals [20,51,52]. We define $y_{i}\left(n\right),i=1,2,\ldots ,L$ as the $i^{th}$ row vector of the extracted matrix Y. The extracted signal $y_{i}\left(n\right)$ is estimated using PCA, FastICA, and NSITM algorithms. We also define $s_{i}\left(n\right)$ as the corresponding $i^{th}$ row vector of the source matrix S, having the same form of Y, as in (6). Then, the SPI is computed as

(24) $$SPI=\frac{1}{L}\sum_{i=1}^{L}10log_{10}\left|\frac{\langle y_{i}\left(n\right),s_{i}\left(n\right)\rangle }{\sqrt{\langle y_{i}\left(n\right),y_{i}\left(n\right)\rangle \langle s_{i}\left(n\right),s_{i}\left(n\right)\rangle }}-1\right|,$$

where L=2 (the FECG and MECG sources), and $\langle .\rangle$ denotes the inner product. To compute SIR, SAR, and SDR, it is required first to decompose the extracted signals $y_{i}\left(n\right)$, as follows:

(25) $$y_{i}\left(n\right)=s_{target}+e_{interf}+e_{noise}+e_{artif},$$

where $s_{target}$ is the component of $s_{i}\left(n\right)$ in $y_{i}\left(n\right)$, $e_{interf}$, $e_{noise}$, and $e_{artif}$ are the interference, noise and artifact error terms, respectively. Second, the terms are computed using BSS EVAL toolbox, as follows [53]:

(26) $$SIR=10log_{10}\frac{{∥s_{target}∥}_{2}^{2}}{{∥e_{interf}∥}_{2}^{2}},$$

(27) $$SAR=10log_{10}\frac{{∥s_{target}+e_{interf}+e_{noise}∥}_{2}^{2}}{{∥e_{artif}∥}_{2}^{2}},$$

(28) $$SDR=10log_{10}\frac{{∥s_{target}∥}_{2}^{2}}{{∥e_{interf}+e_{noise}+e_{artif}∥}_{2}^{2}}.$$

**Figure 17:**
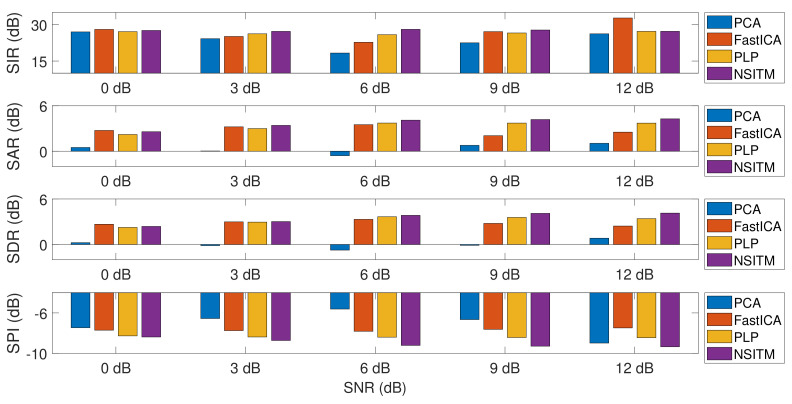
Comparing between the average values of the extraction metrics (SIR, SAR, SDR, and SPI), with the variations of the SNR, using NSTM, FastICA, PCA, and PLP algorithms. The values are taken from Table 2, Table 3 and Table 4. SIR, SAR, and SDR are computed using Equations (26)–(28). SPI is computed using Equation (24).

The simulation is repeated by first fixing the SNR at 0 dB and then varying simulated pregnancy numbers from 1 to 10. For each step, the SPI, SIR, SAR, and SDR are computed, and their average values are obtained. The simulation is repeated by varying the SNR to 3 dB, 6 dB, 9 dB, then to 12 dB. Results of simulation (for only 0 dB, 6 dB, and 12 dB) are recorded in Table 2, Table 3 and Table 4. Results from the proposed NSITM algorithm are provided in bold letters in these tables. The average values of the extraction performances are plotted as shown in Figure 17. Other results (for 3 dB and 9 dB) are not shown due to excessive number of pages. However, they are included in the final results shown in Figure 17. Results from Figure 17 indicate that for SNR equals 0 dB, the proposed NSITM algorithm shows a considerable improvement over others in terms of SPI. However, it shows slightly less value in terms of SIR, SAR, and SDR, as compared with the FastICA. For SNR equals 3 dB, 6 dB, and 9 dB, the proposed NSITM algorithm shows better score, in all metrics, than other algorithms. For SNR equals 12 dB, the proposed NSITM algorithm shows the highest scores in SAR, SDR, and SPI, while its SIR score is the next highest score after the FastICA. These results were recorded based on applying the algorithms on the available data. As a general conclusion, the extraction performances of the proposed NSITM algorithm shows mostly considerable improvement with increasing SNR values, as compared with other algorithms.

### 5.4. Experiment 4: FECG Extraction Metrics Based on Fetal-to-Maternal SNR Variations

**Figure 18:**
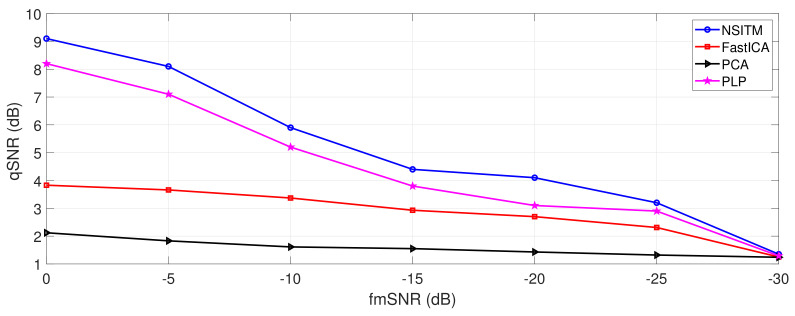
Comparison between qSNR of the proposed NSITM, FastICA, PCA, and PLP algorithms, in terms of fmSNR variations, using Physionet/Fetal ECG Synthetic database (FECGSYNDB). The selected event is maternal heart rate (MHR)/FHR acceleration/deceleration plus noise. The term qSNR is computed by (30).

In this section, the FECG extraction performance is evaluated by varying the fetal-to-maternal SNR (fmSNR) from −30 dB to 0 dB and extracting the FECG signal (using NSITM, PCA, FastICA, and PLP algorithms). We used the same data from Section 5.3, from channels (10, 11, 18, 19) and the two reference channels (33 and 34). First, the FECG and MECG signal are generated as discussed in Section 5.3, and illustrated in Figure 13. Next, we set the value of fmSNR to −30 dB, as a starting value. To satisfy that the value of fmSNR is −30 dB, the FECG signal is multiplied by a factor, denoted by *p*, which is computed according to [26,49]

(29) $$p=\sqrt{\frac{p_{m}}{p_{f}}}.10^{-q/20},$$

where $p_{m}$ and $p_{f}$ are the MECG and FECG signal power, respectively, and q = fmSNR. The mixture signal is then computed by adding the MECG signal to the FECG signal (after multiplying by *p*) [26,49]. This procedure was repeated for all used signals for channels (10, 11, 18, 19). The mixture signals are then passed to the NSITM and other algorithms to extract the FECG and MECG signals. Finally, the quality signal-to-noise ratio (qSNR) of the extracted FECG signal is then computed as [26,54]:

(30) $$qSNR=10log\frac{\sum_{n=1}^{N}{\left({\hat{s}}_{f}^{k}\left(n\right)\right)}^{2}}{\sum_{n=1}^{N}{\left(s_{f}^{k}\left(n\right)-{\hat{s}}_{f}^{k}\left(n\right)\right)}^{2}},$$

where $s_{f}^{k}\left(n\right)$ and ${\hat{s}}_{f}^{k}\left(n\right)$ are the FECG and the extracted FECG signals for channel *k*, k = 10, 11, 18, 19. All above steps were repeated to extract FECG and MECG sigals when fmSNR is −25 dB, −20 dB, …, 0 dB. Figure 18 illustrates a plot of qSNR versus fmSNR variations using the NSITM, PCA, FastICA, and PLP algorithms. Results from Figure 18 show considerable improvement of qSNR using the proposed NSITM, as compared with PCA, FastICA, and PLP, when fmSNR varies from −30 dB to 0 dB.

### 5.5. Experiment 5: Performance Evaluation Using Statistical Measures

In this simulation, we used three statistical measures, the sensitivity (SE), the accuracy (ACC), and the positive predictive value (PPV), in order to evaluate the performance of the proposed algorithm in detecting the FECG peaks [4,7,29,54]. We used the same real ECG Data, from Physionet Challenge 2013 data set a, and the simulation carried out in Section 5.2. Then, the extracted FECG signal is used to compute the SE, ACC, and PPV, as follows:

(31) $$SE\%=\frac{TP}{TP+FN}\times 100\%,$$

(32) $$ACC\%=\frac{TP}{TP+FN+FP}\times 100\%,$$

(33) $$PPV\%=\frac{TP}{TP+FP}\times 100\%,$$

where TP, FN, and FP are true positive, false negative, and false positive, respectively. Results were recorded in Table 5.

From the results in Table 5, it is clear that the the proposed algorithm NSITM scores the highest mean values in SE, ACC, and PPV, as compared with other algorithms. Thus, the proposed NSITM algorithms has resulted in significant improvement in FECG signal detection as compared with other algorithms used in this paper.

## 6. Discussions

### 6.1. Discussion on Experiment 1

In the first experiment, visual FECG and MECG waves are provided to demonstrate the advantages of our proposed algorithm, based on using real ECG data from Daisy database. From Figure 6, Figure 7 and Figure 8, one may gain the following insights:

1. The proposed NSITM algorithm is effective in extracting the FECG and MECG signals from the ECG mixture. The extraction shows some background noise, using the proposed NSITM and all used methods. This requires further investigation and is probably covered in future work.
2. As the ECG signal from Daisy database is clean, the proposed NSITM is able to extract the FECG signal directly form the raw FECG signal obtained from Null(.) stage in Figure 2. This decision is taken by the control logic in Figure 2, and explained in Algorithm 1, step 13.

### 6.2. Discussion on Experiment 2

This experiment is similar to experiment 1, in visualization the FECG and MECG signals. However, real ECG data were provided from the Physionet database. From Figure 9, Figure 10, Figure 11 and Figure 12, we have the following remarks:

1. As the data used in this experiment is noisy, the proposed NSITM algorithm and other algorithms used in this experiment, provide raw FECG signals that contain both FECG and MECG signals, as shown in Figure 10. Thus, the MECG components need to be removed using ACF. First, the MECG signals were extracted as shown in Figure 11. Then, the locations of R peaks in the MECG signal are estimated. These locations are used to adjust the ACF in order to remove the MECG components from the raw FECG signals.
2. The extracted FECG and MECG signals, using the proposed NSITM, are better than other extracted FECG and MECG signals using PCA, FastICA, and PLP algorithms.

### 6.3. Discussion on Experiment 3

In this experiment, visual FECG and MECG signals and four extraction performances (SIR, SAR, SDR, and SPI) are provided to address the effectiveness of our proposed algorithm, based on using synthetic data from Physionet database, considering MHR/FHR acceleration/deceleration plus noise. From Figure 13, Figure 14, Figure 15, Figure 16 and Figure 17 and Table 2, Table 3 and Table 4, one may point out the following remarks:

1. The proposed NSITM algorithm is effective in extracting the FECG and MECG signals from the ECG mixture. As there were no MECG components in the raw FECG signals, the ACF will be deactivated by the control logic and the raw FECG signal is considered as the extracted FECG signal, as shown in Figure 15.
2. As illustrated in Figure 15, the extracted FECG signal using the proposed NSITM is better than other extracted FECG signals using PCA, FastICA, and PLP algorithms.
3. As illustrated in Figure 17, the average values of the extraction performances SIR, SAR, SDR, and SPI are significantly better for the NSITM algorithm than those results obtained using PCA, FastICA, and PLP algorithms, for SNR equal to 3 dB, 6 dB, 9 dB, and 12 dB. However, for SNR = 0 dB, the FastICA shows a slightly better performance. This is due to limited number of data, i.e., subjects, used in the experiment. An increasing amount of experimental data may show better performances using NSITM, as is the case for 3dB, 6 dB, 9 dB, and 12 dB. We used the available data to run this simulation.

### 6.4. Discussion on Experiment 4

In this experiment, the effect of varying the fmSNR on the qSNR is provided to address the effectiveness of our proposed algorithm in extracting the FECG for different values of fmSNR, and based on using synthetic data from experiment 3. From Figure 18, one may point the following remarks:

1. At very low fmSNR, −30 dB, the proposed NSITM algorithm and other algorithms show the same low level of qSNR, which is equal to 1.29 dB. This is expected from all BSS algorithms at very low SNR.
2. As the fmSNR increased, the proposed NSITM shows a considerable qSNR improvement as compared with all other algorithms. The maximum qSNR was recorded to be at 9.1 dB when the fmSNR is 0 dB.
3. The next considerable algorithm is the PLP that shows a qSNR value of 8.2 dB at 0 dB fmSNR.
4. The FastICA and PCA performance scores for the third and the fourth places with qSNR of 3.83 dB and 2.12 dB, respectively, at fmSNR = 0 dB.

### 6.5. Discussion on Experiment 5

In this simulation, three statistical measures (SE, ACC, and PPV) were used to evaluate the performance of the proposed algorithm in detecting the FECG peaks. A real data from experiment 2 were used in this simulation. From Table 5, the followings points may be noted:

1. The proposed NSITM algorithm scores the highest average SE value (99%) as compared with other algorithms. The next highest scores are (98%, 97.3%, and 96.1%), using the PLP, FastICA, and PCA algorithms, respectively.
2. The proposed NSITM algorithm scores the highest average ACC value (97%) as compared with other algorithms. The next highest scores are (95.5%, 93.3%, and 91.9%), using the PLP, FastICA, and PCA algorithms, respectively.
3. The proposed NSITM algorithm scores the highest average PPV value (97.9%) as compared with other algorithms. The next highest scores are (97.4%, 95.7%, and 95.4%), using the PLP, FastICA, and PCA algorithms, respectively.

### 6.6. Future Work

In summary, our best results on the Daisy and Physionet (Challenge 2013 and Synthetic) databases were achieved by using the proposed NSITM as compared with other algorithms, for the majority of used data files. This is the best that we can do due to the absence of large publicly available databases with expert references [7].

Furthermore, the FECG morphological evaluation of both the simulated and real data are in its earliest stages and requires further investigations. However, we have achieved some extraction performances based on synthetic databases.

Furthermore, our proposed NSITM uses ACF to remove the MECG components from the raw extracted FECG. This may cause the removal of some information from the raw FECG signal if the FECG and MECG R peaks are overlapped. Thus, further investigation may be required to remove the MECG component using new post processing techniques.

Furthermore, as the proposed NSITM has less computational complexity as compared with other NCA algorithms. Hence, the algorithm has the potential to be implemented in real time. Further investigation may be required to implement the algorithm for real-time applications that require cooperation with heath care providers and medical doctors.

## 7. Conclusions

A noninvasive FECG extraction algorithm, referred to as NSITM, has been presented. The design problem has been formulated and an analysis has also been provided. The proposed algorithm computes first the ITM matrix W from the original ECG input. Then, the raw FECG and MECG signals are estimated from the Null space of W. The clean FECG signal is then extracted by removing the unwanted MECG component from the raw FECG signal. This requires FECG/MECG peak detection and a decision-making algorithm to address the exact locations of the MECG peaks. The computational complexity of the proposed algorithm have shown considerable improvement as compared with the previous NCA algorithm. The proposed algorithm was simulated using real and synthesised ECG data, and compared with PCA, FastICA, and PLP algorithms. Visual results using (DAISY) real data have shown that the proposed algorithm is effective in extracting FECG and MECG signals, when selecting the number of abdominal signals to be 5, with two reference signals taken from the thorax. Visual results using real data from the Physionet Challenge 2013 dataset/set a have shown the existence of MECG R peaks in the FECG signals. The MECG peaks have been removed using ACF, thus extracting clean FECG signals. The robustness of the proposed algorithm over time was checked to address the effectiveness of the algorithm in extracting the FECG and MECG signals.

Results of applying the NSITM algorithm to the Physionet/Fetal ECG Synthetic database (FECGSYNDB) have shown the capability of the algorithm in extraction FECG and MECG signals from all eight data signals used in simulation, and for all selected SNR values (available from the Physionet database from 0 dB to 12 dB), with MHR/FHR acceleration/deceleration plus noise being selected as the event type. The average values of the extraction performance metrics (SIR, SAR, SDR, and SPI for the NSITM algorithm have mostly shown significant improvement compared to other algorithms, when data files are used with SNR from 0 dB to 12 dB. Results on applying the NSITM algorithm to the same synthetic data have shown considerable improvement in qSNR when fmSNR varied from −30 dB to 0 dB. The proposed algorithm was also evaluated using statistical measures (SE, ACC, and PPV). Results on applying the proposed algorithm on the Physionet Challenge 2013 data/set a have shown the highest statistical values of SE, ACC, and PPV, as compared with other algorithms.

## Figures and Tables

**Table 1:** Examples of how to rename the files downloaded from the FECGSYNDB large database [47], considering SNR = 0 dB, 6 dB, and 12 dB. The paper file name is used in this paper to shorten the long file name from [47]. Its format is XYYZZ, where X is an abbreviation for the synthesized signal, and is equal to F (for FECG), or M (for MECG), or N (for Noise), YY is the simulated pregnancy number (00-10), ZZ is the SNR (00 dB, or 06 dB, or 12 dB). The ‘l1’ code in the downloaded file name refers to the repetition number (1 to 5). It was selected as 1 in this paper.

Simulated Pregnancy Number	SNR	Type of	File Name Used Synthesised Signal in the Paper	File Name Downloaded from [47]
		FECG	F0100	sub01/snr00dB/sub01_snr00dB_l1_fecg1
01	0 dB	MECG	M0100	sub01/snr00dB/sub01_snr00dB_l1_MECG
		Noise	N0100	sub01/snr00dB/sub01_snr00dB_l1_noise1
		FECG	F0506	sub05/snr06dB/sub05_snr06dB_l1_fecg1
05	6 dB	MECG	M0506	sub05/snr06dB/sub05_snr06dB_l1_MECG
		Noise	N0506	sub05/snr06dB/sub05_snr06dB_l1_noise1
		FECG	F1012	sub10/snr12dB/sub105_snr12dB_l1_fecg1
10	12 dB	MECG	M1012	sub10/snr12dB/sub10_snr12dB_l1_MECG
		Noise	N1012	sub10/snr12dB/sub10_snr12dB_l1_noise1

**Table 2:** Comparison between the FECG extraction performances (SPI, SIR, SAR, and SDR), using the proposed NSITM, PCA, FastICA, and PLP algorithms, considering SNR = 0 dB. Data are collected from Physionet/Fetal ECG Synthetic database (FECGSYNDB).

Paper File Names										
**Extraction Metric**	**Algorithm**	**F0100**	**F0200**	**F0300**	**F0600**	**F0700**	**F0800**	**F0900**	**F1000**	**Average**
		**M0100**	**M0200**	**M0300**	**M0600**	**M0700**	**M0800**	**M0900**	**F1000**	
		**N0100**	**N0200**	**N0300**	**N0600**	**N0700**	**N0800**	**N0900**	**N1000**	
	PCA	14.57	25.42	39.81	27.61	28.18	34.37	28.39	17.74	27.01
SIR	FastICA	22.73	26.41	29.26	31.71	27.19	39.38	24.33	23.26	28.03
(dB)	PLP	22.94	27.35	28.85	32.33	21.54	35.45	32.39	16.12	27.12
	**NSITM**	**24.51**	**28.11**	**29.08**	**32.69**	**21.63**	**35.71**	**33.04**	**16.14**	**27.61**
	PCA	−11.91	−0.33	8.86	12.59	3.98	2.81	−12.38	0.36	0.49
SAR	FastICA	−2.41	6.44	5.92	2.57	4.41	2.47	−0.56	3.13	2.74
(dB)	PLP	−2.33	6.45	6.11	4.52	−4.02	2.35	1.25	3.37	2.21
	**NSITM**	**−2.24**	**6.48**	**6.43**	**6.47**	**−3.91**	**2.57**	**1.57**	**3.52**	**2.57**
	PCA	−12.73	−0.38	8.81	12.38	3.82	2.82	−13.01	0.22	0.24
SDR	FastICA	−2.43	6.42	5.81	2.55	4.07	2.41	−0.52	3.03	2.66
(dB)	PLP	−2.39	6.11	5.76	6.12	−4.12	2.17	1.44	2.96	2.25
	**NSITM**	**−2.41**	**6.41**	**6.09**	**6.16**	**−4.11**	**2.24**	**1.57**	**3.12**	**2.38**
	PCA	−1.28	−6.57	−12.56	−10.16	−8.61	−8.01	−6.86	−5.51	−7.40
SPI	FastICA	−5.14	−10.37	−9.71	−7.20	−8.56	−7.58	−5.82	−7.29	−7.70
(dB)	PLP	−5.21	−10.73	−10.22	−9.61	−6.92	−7.94	−8.27	−7.32	−8.27
	**NSITM**	**−5.22**	**−11.03**	**−10.59**	**−10.68**	**−4.12**	**−6.48**	**−11.33**	**−7.61**	**−8.38**

**Table 3:** Comparison between the FECG extraction performances (SPI, SIR, SAR, and SDR), using the proposed NSITM, PCA, and FastICA algorithms, considering SNR = 6 dB. Data are collected from Physionet/Fetal ECG Synthetic database (FECGSYNDB).

Paper File Names										
**Extraction Metric**	**Algorithm**	**F0103**	**F0203**	**F0303**	**F0603**	**F0703**	**F0803**	**F0903**	**F1000**	**Average**
		**M0103**	**M0203**	**M0303**	**M0603**	**M0703**	**M0803**	**M0903**	**F1030**	
		**N0103**	**N0203**	**N0303**	**N0603**	**N0703**	**N0803**	**N0903**	**N1030**	
	PCA	17.56	19.81	15.62	12.37	25.33	16.41	19.23	20.12	18.31
SIR	FastICA	8.14	27.24	19.33	26.12	26.38	20.93	22.11	21.79	21.51
(dB)	PLP	12.41	32.25	19.52	29.91	30.24	21.03	38.77	22.83	25.87
	**NSITM**	**14.56**	**36.71**	**18.91**	**33.13**	**32.49**	**22.18**	**41.74**	**24.82**	**28.07**
	PCA	−13.81	−0.96	3.07	7.24	5.82	−3.31	−1.89	−0.83	−0.58
SAR	FastICA	1.92	4.56	5.08	2.39	5.88	3.62	5.52	−0.93	3.51
(dB)	PLP	−2.03	4.36	4.93	4.37	6.26	4.15	6.52	1.22	3.72
(dB)	**NSITM**	**−2.17**	**4.23**	**4.22**	**6.31**	**6.77**	**5.06**	**6.92**	**1.47**	**4.10**
	PCA	−13.77	−1.62	3.05	7.11	5.77	−3.53	−2.12	−0.84	−0.74
SDR	FastICA	1.12	4.55	5.09	2.35	5.81	3.28	5.24	−0.92	3.31
(dB)	PLP	−1.23	4.44	3.59	4.71	6.15	4.27	6.15	1.24	3.66
	**NSITM**	**−3.17**	**4.23**	**3.67**	**6.19**	**6.75**	**4.81**	**6.82**	**1.41**	**3.84**
	PCA	−1.67	−6.43	−7.91	−9.29	−10.11	−3.39	−4.07	−2.12	−5.62
SPI	FastICA	−6.22	−9.21	−9.58	−7.01	−9.62	−8.36	−9.47	−3.11	−7.82
(dB)	PLP	−5.46	−9.31	−9.42	−9.55	−10.32	−8.89	−10.84	−3.44	− 8.40
(dB)	**NSITM**	**−4.59**	**−9.52**	**−9.32**	**−11.49**	**−10.72**	**−10.77**	**−13.69**	**−3.61**	**−9.21**

**Table 4:** Comparison between the FECG extraction performances (SPI, SIR, SAR, and SDR), using the proposed NSITM, PCA, and FastICA algorithms, considering SNR = 12 dB. Data are collected from Physionet/Fetal ECG Synthetic database (FECGSYNDB).

Paper File Names										
**Extraction Metric**	**Algorithm**	**F0112**	**F0212**	**F0312**	**F0612**	**F0712**	**F0812**	**F0912**	**F1012**	**Average**
		**M0112**	**M0212**	**M0312**	**M0612**	**M0712**	**M0812**	**M0912**	**F1012**	
		**N0112**	**N0212**	**N0312**	**N0612**	**N0712**	**N0812**	**N0912**	**N1012**	
	PCA	16.09	22.78	33.43	19.81	27.28	41.57	22.53	26.34	26.23
SIR	FastICA	25.66	39.81	33.41	49.47	36.31	28.67	15.51	33.12	32.74
(dB)	PLP	3.42	32.37	28.11	36.52	24.35	18.34	24.31	20.42	27.23
	**NSITM**	**36.56**	**30.87**	**29.81**	**28.93**	**26.91**	**18.68**	**24.91**	**21.32**	**27.25**
	PCA	−10.24	−0.64	3.01	10.84	7.03	7.02	−11.76	2.78	1.05
SAR	FastICA	0.24	6.51	7.93	4.97	5.67	0.23	−6.51	1.11	2.52
(dB)	PLP	0.12	6.03	8.13	7.63	5.57	0.46	−1.53	3.27	3.71
	**NSITM**	**0.099**	**5.97**	**8.33**	**9.66**	**5.59**	**0.58**	**−0.39**	**4.35**	**4.27**
	PCA	−10.34	−0.66	2.99	9.9	7.12	7.01	−12.11	2.73	0.83
SDR	FastICA	0.21	6.49	7.91	4.97	5.68	0.15	−7.07	1.13	2.43
(dB)	PLP	0.15	6.12	8.32	6.49	5.51	0.27	−2.62	3.08	3.41
	**NSITM**	**0.089**	**5.91**	**8.23**	**9.51**	**5.43**	**0.38**	**−0.62**	**4.18**	**4.14**
	PCA	−1.62	−6.35	−11.41	−13.81	−11.01	−10.78	−9.72	−7.18	−8.98
SPI	FastICA	−2.16	−10.62	−11.43	−8.93	−9.49	−7.07	−4.28	−5.93	−7.48
(dB)	PLP	3.94	−10.62	−11.61	−12.78	−9.51	−5.69	−6.15	−7.32	−8.45
	**NSITM**	−5.92	−10.61	−12.61	−14.21	−9.57	−5.71	−7.07	−9.11	−9.35

**Table 5:** Statistical measures on data collected from Physionet/Computing in Cardiology Challenge 2013 database obtained by the proposed NSITM, PCA, FastICA, and PLP algorithms.

Algorithm	File Number	Detected Peaks	TP	FP	FN	SE (%)	ACC (%)	PPV (%)
	a04	131	126	5	4	96.9	93.3	96.2
	a08	130	122	7	6	95.3	90.4	94.6
PCA	a14	131	124	7	6	95.4	90.5	94.7
	a15	131	125	6	5	96.2	91.9	95.4
	a25	131	126	5	4	96.9	93.3	96.2
				**Mean values**	→	**96.1**	**91.9**	**95.4**
	a04	130	126	4	3	97.7	94.7	96.9
	a08	130	123	7	4	96.9	91.8	94.6
FastICA	a14	130	124	6	3	97.6	93.2	95.4
	a15	130	124	6	4	96.9	92.5	95.4
	a25	131	126	5	3	97.7	94.0	96.2
				**Mean values**	→	**97.3**	**93.3**	**95.7**
	a04	130	127	3	3	97.7	95.5	97.7
	a08	130	126	4	4	96.9	94.0	96.9
PLP	a14	130	128	2	1	99.2	97.7	98.5
	a15	130	127	3	2	98.4	96.2	97.7
	a25	131	126	5	3	97.7	94.0	96.2
				**Mean values**	→	**98.0**	**95.5**	**97.4**
	a04	130	127	3	2	98.4	96.2	97.7
	a08	131	128	3	2	98.5	96.2	97.7
NSITM	a14	130	129	1	0	100	99.2	99.2
	a15	130	127	3	1	99.2	96.9	97.7
	a25	131	127	4	1	99.2	96.2	96.9
				**Mean values**	→	**99.1**	**97.0**	**97.9**
